# Diversification of HP1‐like Chromo Domain Proteins in *Tetrahymena thermophila*

**DOI:** 10.1111/jeu.12443

**Published:** 2017-08-03

**Authors:** Emily A. Wiley, Scott Horrell, Alyssa Yoshino, Cara C. Schornak, Claire Bagnani, Douglas L. Chalker

**Affiliations:** ^1^ W.M. Keck Science Center of Claremont McKenna, Pitzer, and Scripps Colleges Claremont California 91711; ^2^ Department of Biology Washington University St. Louis Missouri 63130

**Keywords:** Chromatin structure, heterochromatin protein, heterochromatin structure, macronucleus, micronucleus

## Abstract

Proteins that possess a chromo domain are well‐known for their roles in heterochromatin assembly and maintenance. The Heterochromatin Protein 1 (HP1) family, with a chromo domain and carboxy‐terminal chromo shadow domain, targets heterochromatin through interaction with histone H3 methylated on lysine 9 (H3K9me2/3). The structural and functional diversity of these proteins observed in both fission yeast and metazoans correlate with chromatin specialization. To expand these studies, we examined chromo domain proteins in the ciliate *Tetrahymena thermophila*, which has functionally diverse and developmentally regulated heterochromatin domains. We identified thirteen proteins similar to HP1. Together they possess only a fraction of the possible chromo domain subtypes and most lack a recognizable chromo shadow domain. Using fluorescence microscopy to track chromatin localization of tagged proteins through the life cycle, we show evidence that in *T. thermophila* this family has diversified with biological roles in RNAi‐directed DNA elimination, germline genome structure, and somatic heterochromatin. Those proteins with H3K27me3 binding sequence characteristics localize to chromatin in mature nuclei, whereas those with H3K9me2/3 binding characteristics localize to developing nuclei undergoing DNA elimination. Findings point to an expanded and diversified family of chromo domain proteins that parallels heterochromatin diversity in ciliates.

THE “CHRomatin Organization MOdifier” or CHROMO domain (CD) is a 40–55 amino acid domain found on a variety of proteins involved with chromatin structure formation, stability, remodeling, and gene expression regulation in eukaryotes. The protein families that contain CDs include the Chromo domain‐Helicase‐DNA‐binding (CHD) family, heterochromatin protein 1 (HP1) family, Polycomb (Pc) family, methyltransferase family, Msl‐3 family of transcription regulators, SWI3 chromatin remodeling subunits, the histone acetyltransferase family, and ankyrin family (Tajul‐Arifin et al. 2003; reviewed in Eissenberg 2012). Originally identified on HP1 and Pc family proteins (Paro and Hogness 1991) the CD mediates chromatin interactions by binding post‐translational modifications on the histone H3 N‐terminus (Jacobs et al. 2001; Stewart et al. 2005; reviewed in Eissenberg 2012). There is diversity in these binding modules for methylated histone H3, for example: the CDs on HP1 homologs exhibit specificity for binding histone H3 di‐ or tri‐methylated at lysine 9 (H3K9me2/3), CDs on Pc homologs show higher affinity for tri‐methylated lysine 27 (H3K27me3), and CDs of CHD family members bind H3K4me, common at transcriptional start sites (Bernstein et al. 2005; Fischle et al. 2003; Liang et al. 2004; Schübeler et al. 2004). The interaction with methylated H3 lysines K9 and K27 in heterochromatin requires three aromatic amino acids within the CD, known as the “aromatic cage” (Jacobs and Khorasanizadeh 2002; Nielsen et al. 2002); mutation of any one of these results in loss of heterochromatin localization (Platero et al. 1995). Interactions of CD‐containing proteins with chromatin may also be mediated by direct binding of DNA (Bouazoune et al. 2002), or through recognition of RNA‐chromatin complexes, with some CDs serving as RNA interaction module (Akhtar et al. 2000; Bernstein et al. 2006; Muchardt et al. 2002).

Many CD proteins, primarily those in the HP1 family, contain an additional “chromo shadow domain” (CSD). Typically located at the C‐terminus, the CSD mediates homo‐dimerization and interactions with other proteins such as histone methyltransferases to maintain higher order heterochromatin structures (Aasland and Stewart 1995; Brasher 2000; Schotta et al. 2002). There are structural differences between CDs and CSDs including a putative protein interaction pit at the dimer interface of CSD's that may provide another means of targeting to chromatin domains (Cowieson et al. 2000), and indeed some CSDs alone are able to target heterochromatin (Smothers and Henikoff 2001). Moreover, single amino acid changes in the CSD domain of the archetype HP1 homolog in *Drosophila* (dHP1a) alter its ligand specificity (Mendez et al. 2011, 2013), highlighting the importance of the CSD for HP1 protein targeting and function.

Heterochromatin structures are complex and a diverse array of chromatin modifications and regulatory proteins are required to regulate these structures within the genome (Riddle et al. 2011). The HP1 and Polycomb protein families are best known for their roles in heterochromatin formation, epigenetic silencing, and genome organization (Cubeñas‐Potts and Corces 2015; Eissenberg and Elgin Sarah 2014), and great expansion and diversification of the HP1 family may correlate with functional diversification of heterochromatin in *Drosophila* species (Levine et al. 2012; Riddle et al. 2011). Among the protozoans, the ciliate *Tetrahymena thermophila* provides opportunity to further explore heterochromatin protein diversity and function. *Tetrahymena* cells possess two structurally and functionally distinct nuclei. The macronucleus is transcriptionally active throughout the life cycle and contains both euchromatin and heterochromatin, whereas the germ line micronucleus is transcriptionally silent—all chromosomes are compacted into heterochromatin‐like structures. During sexual conjugation, both the macro‐ and micronucleus are produced from division of one zygotic nucleus. Subsequent differentiation of the two nuclei involves extensive genome rearrangements resulting in the elimination of 50 Mbp from thousands of loci in the developing new macronucleus. These internal eliminated sequences (IESs) are targeted by small RNA‐directed heterochromatin formation that involves marking these loci with H3K27 and H3K9 methylation (Liu et al. 2004, 2007; Taverna et al. 2002). Once established, this heterochromatin is bound by HP1‐like proteins Pdd1 and Pdd3 (Coyne et al. 1999; Madireddi et al. 1996; Nikiforov et al. 2000). In parallel, the old (parental) macronucleus degrades by an apoptosis‐like mechanism involving whole genome condensation and fragmentation (Davis et al. 1992; Mpoke and Wolfe 1996). The micronucleus must also be restructured during sexual conjugation as it decondenses then recondenses prior to meiosis coincident with brief transcriptional activity (Martindale et al. 1982).

The ability to synchronize the dynamic chromatin changes that occur during sexual development and nuclear differentiation within a *Tetrahymena* cell population presented an opportunity to gain unique insight into the functional diversity of CD‐containing heterochromatin proteins. We describe an expanded and diversified family of thirteen HP1‐like proteins that contain a limited number of CD subtypes. Their localization to different developmentally regulated regions of heterochromatin with different biological roles points to diversified chromatin functions within this family and variable roles for the CSD in chromatin targeting.

## Materials and Methods

### Sequence analysis

Chromo domain proteins in the *Tetrahymena* genome were first identified by BLAST searching the *Tetrahymena* protein database with the amino acid sequence from *Drosophila* Hp1a CD. The amino acid sequences of identified *Tetrahymena* CD proteins were analyzed using BLASTP (www.ncbi.nlm.nih.gov), Pfam 30.0 (http://pfam.xfam.org), and SMART (http://smart.embl-heidelberg.de) to determine the CD boundaries on each. Sequence alignment comparisons of the CD sequences were performed using Multiple Sequence Alignment‐CLUSTALW [EMBL‐EBI, Welcome Trust Genome Campus, Cambridgeshire, U.K. (http://www.ebi.ac.uk/Tools/msa/clustalw2/)]. The CLUSTAL protein alignment was performed using a gap open penalty of 10, a gap extension penalty of 0.05, a hydrophobic gap, no weight transition, and a BLOSUM weight matrix. Molecular phylogenetic relationships were computed by first aligning sequences by Multiple Sequence Comparison by Log‐Expectation (MUSCLE) using default parameters. Output in Pearson/FASTA format was analyzed using maximum likelihood (PhyML 3.0; http://phylogeny.lirmm.fr/phylo_cgi/index.cgi) with the EX2 substitution model (Dereeper et al. 2008; Edgar 2004). Branch support was computed using SH‐like Approximate Likelihood Ratio tests.

To analyze CD subtypes, the Tetrahymena Genome Database (http://ciliate.org) was searched with published HMM models for the 26 previously characterized CD subtypes (Tajul‐Arifin et al. 2003). All *Tetrahymena* protein matches with E‐values less than or equal to 10^−3^ were considered candidates for containing that particular subtype. Chromo domain subtype assignments for a protein were made based on which of the 26 subtypes matched with the lowest E‐value. Visual inspection confirmed that the subtype with the most conserved “invariable” residues was assigned. The same procedure was used to assign CD subtypes to the second CD, called “CD2” (in the amino to carboxyl direction on the peptide), on Hpl2 and Pdd1 (others failed to be identified by this method). We then used these CD2 amino acid sequences to search all *Tetrahymena* proteins and found matches to second CDs with E‐values less than 10^−3^ on Hpl2, Hpl4, and Hpl7, and Cdl3.

### Strains and cell culture conditions

*Tetrahymena thermophila* strains B2086 (*mat1‐2/mat1‐2* (*mat1‐2*; II) and CU428 (*mpr1‐1/mpr1‐1 MPR1*; mp‐s, VII) provided by the National Tetrahymena Stock Center at Cornell University, were used as wild‐type strains. For all experiments, *T. thermophila* strains including those expressing GFP‐ or YFP‐fused proteins and mutant variants were grown in Super Proteose Peptone medium (2% proteose peptone, 0.1% yeast extract, 0.2% glucose, and 0.003% sequestrine) containing 1× PSF (Penicillin, Streptomycin, and Fungizone; Gibco‐BRL, Gaithersburg, MD) with shaking (70–100 rpm) at 30 °C, until mid‐logarithmic phase (1 × 10^5^ to 3 × 10^5^ cells/ml). For cell starvation, cells were washed once and then suspended, both in 10 mM Tris–HCl (pH 7.4) at a density of 3 × 10^5^ cells/ml, then incubated for 15–18 h at 30 °C without shaking. To conjugate cells, cells (B2086 and CU428, or strains expressing GFP‐ or YFP‐fused proteins) were first starved, then mixed in equal cell numbers in petri dishes and incubated at 30 °C without shaking in a moist chamber.

### Expression and imaging of HPL‐YFP fusions

The coding regions of HPL genes were amplified from genomic DNA by PCR and inserted into pENTR/D by topoisomerase mediated cloning reactions (Invitrogen/Life Technologies, Carlsbad, CA), then transferred into pICY‐gtw by LR Clonase II recombination as previously described (Malone et al. 2008). Oligonucleotides used to amplify each are listed in Table 1. These YFP expression constructs were introduced into *Tetrahymena* cells by conjugative electroporation (Gaertig and Kapler 2000; Gaertig et al. 1994). Transformants were selected and propagated in medium containing 100 μg/ml paromomycin (Sigma‐Aldrich, St. Louis, MO). Expression of Hpl‐YFP proteins was induced by addition of 0.5–1 μg/ml CdCl_2_ in growing cells or 0.03–0.1 μg/ml in mating cells. To prepare for mating, cells were then washed, starved (11–12 h at 30 °C), and mixed in 10 mM Tris medium with wild‐type strains CU428 or B2086. For epifluorescence microscopy, cells were harvested by low‐speed centrifugation (1,000 *g*) and immobilized in ~6 μl of 2% methylcellulose. Slides were viewed under a 60X oil immersion lens on a Nikon Eclipse E600 upright microscope. Differential interference contrast (DIC) and YFP fluorescence images were captured using a Qimaging RetigaEX charge‐coupled‐device camera (Burnaby, BC, Canada) and Openlab software (PerkinElmer, San Jose, CA).

**Table 1:** Oligonucleotides used to amplify HPL coding regions (5′–3′)

HPL1	
UCP1TopoF	CACCATGGCTAAGATAAAATACGAAGGAAGTC
UCP1TopoR	GATATCGATATTTATTCTATTTTCTTTCCCGTTTACC
HPL4	
HP4COTopoF	CACCATGGAACAATAATAAACAAATTTAGAATACTC
HP4COTopoR	GATATCATTAAGAAATTTATTTTATAGGATTTAATCAATG
HPL5	
CP1COTopoF	CACCATGAATAACAAATATTATCATCAACCTTCC
CP1COTopoR	GATATCTTTTTAACTTTTATCTATTTCATTATTAATTTAATTTG
HPL6	
EBS_245410_UP	CACCattaaaATGAGTGACACAACAACAACA
EBS_245410_DS	GATATCCTTTCTTTTTAAGGCTTTGTCTTCA
HPL7	
KAB_551070_UP	CACCtcaaacATGAAAAAGAGAAGACAATC
KAB_551070_DS	GATATCCATTTCTTCCATTTGATTGTTGTTGA

### Expression of chromoshadow domain mutants fused with GFP

The C‐terminal truncations of the *HHP1*,* HPL1*, and *HPL2* genes were created by PCR using *Tetrahymena* genomic DNA and Phusion polymerase (New England Biolabs, Ipswich, MA). Amplified product was directionally inserted into pENTR/D‐TOPO (Invitrogen/Life Technologies) to make plasmids pENTR‐*HHP1.csd;* pENTR*‐HPL1.csd; and* pENTR*‐HPL2.csd*, respectively. Oligonucleotides used to amplify each gene are listed in Table 2. The truncated gene sequences were each confirmed by Sanger sequencing using M13‐F and M13‐R oligonucleotides, then transferred to pIGF‐gtw by LR Clonase II recombination as previously described (Yale et al. 2016). The resulting *pIGF‐GTW::HHP1.csd; pIGF‐GTW::HHP1.csd;* and *pIGF‐GTW::HHP1.csd* plasmids contained the *HHP1, HPL1,* and *HPL2* genes fused at their amino terminus to GFP, under transcriptional control of the *MTT1* promoter. These GFP fusion constructs were transformed into conjugating CU428 x B2086 strains by conjugative electroporation according to a previously published method (Gaertig and Kapler 2000; Gaertig et al. 1994). Transformants were selected and propagated in medium contain 100 μg/ml paromomycin (Sigma‐Aldrich). Expression of the fusion proteins was induced by incubating cultures of *Tetrahymena* transformants for 2 h in CdCl_2_ (2 μg/ml for growing cells; 0.2 μg/ml for starved and conjugating cells). For epifluorescence microscopy, cells were harvested by low‐speed centrifugation (1,000 *g*), the pellet was incubated for 5 min with 0.1 μg/ml of 4′,6 diamino‐2‐phenylindole dihydrochloride (DAPI; Sigma Chemicals), and cells from the pellet were immobilized in ~6 μl of 2% methylcellulose under a #1.5 micro coverslip. Epifluorescence imaging was performed on an upright Leica DM4000 B LED fluorescence microscope with 100X magnification. Images were captured using Leica software (Leica Microsystems, Buffalo Grove, IL).

**Table 2:** Oligonucleotide primers used to amplify CSD truncations of HHP1, HPL1, and HPL2 (5′–3′)

HHP1Δcsd	
HHP1csd_F	CACCATGACAAAAGTTTACGAAGT
HHP1csd_R	TCACTTCACTGGATCTAATGCTT
HPL1Δcsd	
HPL1csd_F	CACCATGGCTAAGATAAAATACGAAG
HPL1csd_R	TCAATTCGTTTTCCAGAACTGGA
HPL2Δcsd	
HPL2csd_F	CACCATGTTCACTGTAAAGCAACAG
HPL2csd_R	TCAAAGATCTTGTAGAGTAGA

### Indirect immunofluorescence microscopy

Growing and conjugating cells were fixed in 2% paraformaldehyde and processed for immunofluorescence as previously described (Cole and Toshiro 2012). Fixed cells were incubated with α‐H3K27me3 antiserum (1:500; Active Motif, #39155) in 1% bovine serum albumin (BSA)/phosphate buffered‐saline (PBS)/0.1% Tween20 (PBST). Cells were then washed 3 × 10 min in PBS, and incubated in secondary antiserum (rhodamine‐conjugated goat anti‐rabbit immunoglobulin G (1:100; Jackson ImmunoResearch #111‐025‐003) in PBST for 1 h at 37 °C, and washed 3 × 15 min in PBS. Fixed cells were counterstained by incubation with 0.1 μg/ml DAPI in 0.1% bovine serum albumin–phosphate‐buffered saline for 10 min. Cells were mounted by adding 5 μl of Vectashield Mounting Medium (Vector Laboratories Inc, Burlingame, CA.) to the cell surface before laying a coverslip over the sample. Epifluorescence imaging of the slides was performed on a Leica DM4000 B LED fluorescence microscope with 100X magnification.

## Results

### Diversity of *Tetrahymena* chromo domain sequences

**Figure 1:**
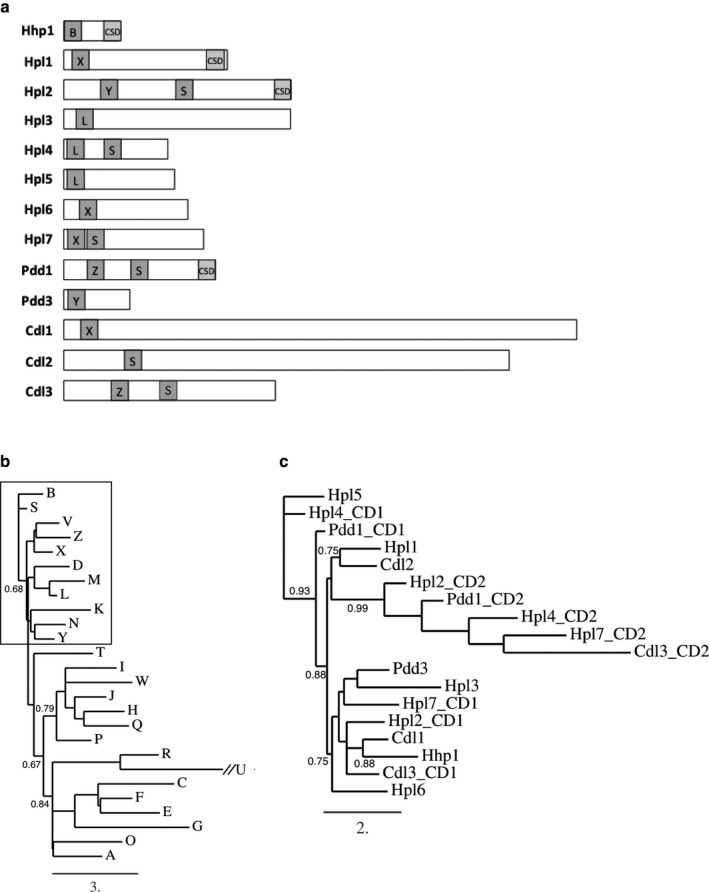
Structures and relationships of *Tetrahymena* Hp1‐like proteins. (**a**) Relative size of the proteins and positions of chromo domains (“CD”, dark gray boxes) on each protein. Chromoshadow domains are represented with light gray boxes and “CSD”. Letters in dark gray boxes represent the CD subtype using the previously published letter designation (Tajul‐Arifin et al. 2003). (**b**) Phylogenetic relationships between HMM models for CD subtypes. Box includes the subset of CD subtypes found on *Tetrahymena* proteins. (**c**) Phylogenetic relationships between CD sequences on the *Tetrahymena* proteins. The CD closest to the amino‐terminus is denoted “CD1” the other (if applicable) is denoted “CD2”.

To identify CD‐containing proteins potentially involved in chromatin condensation and transcriptional silencing, we searched the *T. thermophila* genome for predicted proteins that share similarity to the CD sequence from the founder *Drosophila* HP1 protein, dHP1a, as defined by EMBL‐EBI Pfam (Finn et al. 2016). This strategy identified 15 *Tetrahymena* proteins with putative CDs. Two of these are predicted to also contain helicase domains that are characteristic of the CHD family of chromatin remodeling enzymes and were not further studied. Amino acid sequences for CDs on the remaining thirteen proteins were obtained through comparing predictions from SMART and Pfam 30.0 databases and selecting the consensus sequences. Amino acid sequences for CSDs were obtained through sequence analysis using HHPRED (Soding et al. 2005), which identifies signature domain folds. Of the thirteen putative HP1‐like proteins, four [Hhp1, Hpl1, Hpl2 (*alias* Tcd1), Pdd1] possessed both CD and CSD signature motifs for full‐length HP1 proteins. Two of these four, Hpl2 (Xu et al. 2015) and Pdd1 (Madireddi et al. 1996), are known heterochromatin proteins that have a second CD making them unique from HP1 proteins described in other organisms (Fig. 1a). Hhp1, with similarity to dHP1a through a single CD and CSD is also a known heterochromatin protein (Huang et al. 1998). Nine of the thirteen CD proteins did not possess a recognizable CSD and thus may be considered “partial” HP1 proteins, and three of these partials possessed two full CD's (Fig. 1a).

Analysis of CD sequences from a broad range of organisms has identified 26 CD sub‐types that are present within a variety of protein families including chromatin modifiers, transcription factors, and cytoskeletal‐associated proteins such as ankyrin (Tajul‐Arifin et al. 2003). To determine the CD subtypes on the *Tetrahymena* proteins we searched the Tetrahymena Genome Database with previously published Hidden Markov model (HMM) sequences of the 26 CD subtypes, then inspected each match with an E‐value less than 10^−4^ for conserved residues to assign the best classification. We found that all *Tetrahymena* CDs were between 51 and 55 amino acids in size and could be assigned to six subtype profiles: B, L, S, X, Y, Z (Fig. 1a). Phylogenetic relationships between the 26 HMM profiles revealed that most of these six subtypes are contained within an early‐diverging clade (Fig. 1b). On those proteins containing two CDs, the second (closer to carboxyl terminus) had weaker CD homology, and all of these were classified as subtype S, which is commonly found on proteins containing two CDs in a range of other organisms (Tajul‐Arifin et al. 2003). Subtype B, found only on Hhp1, is a signature of Polycomb (Pc) family proteins and is thus consistent with previous characterization of Hhp1 as having a Pc‐type CD that likely targets H3K27me3 (Yale et al. 2016). Analysis of proteins in well‐studied eukaryotes (*Saccharomyces cerevisiae, Schizosaccharomyces pombe, Drosophila melanogaster, Arabidopsis thaliana, Caenorhabditis elegans,* human, and mouse), revealed that subtype X is commonly found on histone methyltransferases (HMTs) containing a SET domain flanked by pre‐ and post‐SET domains, and subtype L is found on enoyl‐coA hydratase and ankyrin family members (Tajul‐Arifin et al. 2003). Subtypes Y and Z were not found in proteins within this group of commonly studied organisms.

Phylogenetic analysis revealed a robust relationship between the second (minor) CDs (“CD2”) from all proteins that contained two CDs, consistent with their classification as all subtype S (Fig. 1c). Strong similarity was also revealed between the primary CDs of Hpl4 and Hpl5 (both subtype L). The genes encoding these two proteins are located immediately adjacent to each other in the genome, a configuration that presumably arose by gene duplication (Tetrahymena Genome Database). Another clade comprises Hpl2, Cdl1, Hhp1, and Cdl3 (with B, X, Y, and Z subtypes). Another with weaker branch support included Pdd3, Hpl3, Hpl7 (with CD subtypes Y, L, and X respectively). Hpl1 (subtype X), and Cdl2 (subtype S) grouped together with moderately strong branch support (Fig. 1c). The fact that not all the same subtype grouped in the same clade suggests that other sequence elements are better determinants of relationships than highly conserved amino acids in the HMM subtype models that distinguish the different subtypes.

Chromo domains from the well‐studied HP1 and Pc families in various organisms show differences in their binding affinities for methylated lysine 9 on histone H3 (H3K9me2/3) vs. methylated lysine 27 (H3K27me3). Hp1 family CDs generally have higher specificity for di‐ or tri‐methylated K9Me (H3K9me2/3), while Pc CDs interact more broadly with H3K9me2/3 and H3K27me3, and in most cases have stronger interactions with the latter (Bernstein et al. 2005; Fischle et al. 2003; Liang et al. 2004; Schübeler et al. 2004). This difference is attributed in part to more basic character of Pc CDs vs. more acidic for Hp1 CDs (Kaustov et al. 2011). Comparing the overall acidity/basicity of *Tetrahymena* CD sequences revealed that the primary CDs of Hhp1, Cdl3, Hpl6, Hpl4, and Cdl1 were more basic, while Hpl1, 2, 5, 7, Pdd1, Pdd3, and Cdl2 were more acidic similar to Hp1‐type proteins with higher affinity for H3K9me2/3 (Table 3). In fact, it was previously shown that, biochemically, Pdd1 and Pdd3 both bind H3K9me2/3, and Pdd1 can additionally bind H3K27me3 (Liu et al. 2007; Taverna et al. 2002).

**Table 3:** Acidity characteristics of *Tetrahymena thermophila* chromo domains

Protein^a^	ac/ba^b^	pI
Hhp1	0.50	9.6
Cdl3 CD1	0.70	9
Hpl7 CD2	0.75	9.3
Cdl3 CD2	0.75	9
Hpl6	0.80	9
Hpl4 CD1	0.88	8.3
Cdl1	0.88	8.2
Hpl3	1.00	6.4
Pdd1 CD1	1.10	5.8
Pdd3	1.10	5.8
Hpl7 CD1	1.14	5.7
**dHP1a** c	**1.10**	**5.4**
Hpl2 CD1	1.33	4.9
Hpl5	1.38	4.7
Hpl1	1.43	4.8
Hpl4 CD2	2.00	4.4
Pdd1 CD2	2.20	4.2
Cdl2	2.40	4.2
Hpl2 CD2	2.67	4.3

a “CD1” refers to the CD closest to the N‐terminus on proteins with two CDs. “CD2” denotes the second domain.

b Ratio of acidic/basic amino acids.

c *Drosophila* HP1a (dHP1a) is included as a reference (bold).

**Figure 2:**
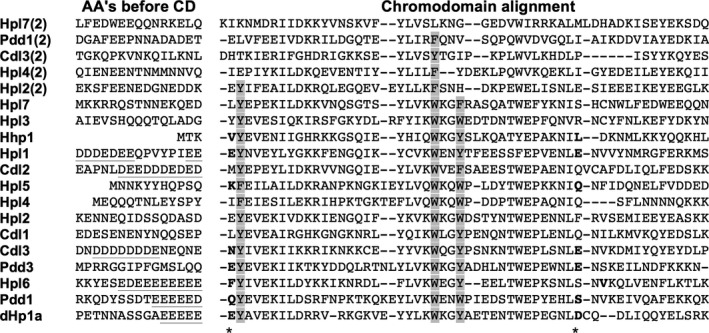
Chromo domain sequence characteristics. CDs were aligned using Clustal Multiple Sequence Alignment by MUSCLE (3.8). Second CDs (from amino terminus) are designated “(2)” next to the protein name. Caging amino acids are shaded, and polar or nonpolar pairs of amino acids common to Hp1‐type or Pc‐type chromo domains, respectively, are bolded and their positions denoted with an asterisk. Acidic N‐terminal extensions are underlined. *Drosophila* Hp1a (denoted “dHp1”) is included as reference.

The nature of amino acids at two other positions (Fig. 2, asterisks) were also found to be significant determinants of binding specificity: one immediately preceding the first caging amino acid and the other 14 amino acids after the last (Kaustov et al. 2011). Nonpolar residues at these positions form a nonpolar “clamp” important for permitting H3K27me binding, whereas polar residues at these positions were necessary for H3K9Me specificity. Examination of *Tetrahymena* CDs revealed a nonpolar clamp on Hpl6 and Hhp1 (Fig. 2, bold) consistent with previous evidence suggesting that Hhp1 binds H3K27me3 (Yale et al. 2016). Residues that could form a polar clamp and have specificity for H3K9Me were found within primary CDs of five proteins: Pdd1, Pdd3, Cdl3, Hpl5, Hpl1. This finding is consistent with data showing that Pdd1 and Pdd3 bind H3K9me2 in vitro (Liu et al. 2007; Taverna et al. 2002).

HP1 family members often contain an N‐terminal acidic patch immediately adjacent to the CD. This patch is proposed to cooperate with nearby phosphorylation to enhance binding of methylated H3 (Shimojo et al. 2016). Examining the 15 amino acids adjacent to the start of the primary CD, a similar acidic patch feature was found on four of the *Tetrahymena* proteins (percentage of acidic residues in patch): Pdd1 (53%), Hpl6 (73%), Cdl2 (73%), and Cdl3 (73%) (Fig. 2, underlined). Hpl1 (60%) had a distinctive acidic patch, but it was located farther from the CD.

Five of the CD‐containing proteins (Hpl2, Hpl4, Hpl7, Cdl3, and Pdd1) have two CD‐related sequences. In each case, the most N‐terminal domain has the greatest similarity to the HP1a CD. Closer examination of the weaker homology domains (CD2) reveal that all have lost at least one of the aromatic caging amino acids critical for binding to methyl‐lysine (Jacobs and Khorasanizadeh 2002; Platero et al. 1995). This finding suggests that the second CD is probably not contributing to binding methylated H3K9 or H3K27.

### Diversity in localization of CD proteins

To examine chromatin localization of the 13 CD proteins most similar in sequence to HP1, each gene was C‐terminally fused to YFP and exogenously expressed in wild type *Tetrahymena* cells. Cdl1,2,3 and Hpl3 were omitted from this analysis due to their large size and lack of CSD domain that reduced their similarity to HP1 (Fig. 1a). Also omitted from the analysis were several proteins whose localization characteristics were previously published (see Table 4 for a summary).

**Table 4:** Summary of expression and localization of *Tetrahymena thermophila* chromo domain proteins

	Expression^a^	Nucleus^b^	Localization description	CD type	References
Hhp1	Gr; conj: hours 6, 16	Mac, anlagen	Chromatin bodies	B	Huang et al. (1998) and Huang et al. (1999)
Hpl1	Conj: hour 9	Anlagen	Pdd1‐marked large foci	X	This study; Kataoka and Mochuziki (2015)
Hpl2	Conj: hour 9	Anlagen	Pdd1‐marked large foci; co‐localization with H3K9Me	Y, S	Xu et al. (2015); S. Horrell and D. Chalker, unpublished data
Hpl4	Conj: hour 9	Anlagen, OM	Large foci in anlagen; uniform in OM	L, S	This study
Hpl5	Conj: hour 9	Anlagen	Uniform	L	This study
Hpl6	Gr; conj: hour 6	Mac	Small foci (sim to Hhp1)	X	This study
Hpl7	Conj: hour 2	mic, anlagen	mic only through early conjugation, foci in anlagen later	X, S	This study
Pdd1	Conj: hour 2	Anlagen, OM	DNA elimination foci in anlagen; binds H3K9Me & H3K27Me	Z, S	Madireddi et al. (1996), Taverna et al. (2002), Liu et al. (2007), and Schwope and Chalker (2014)
Pdd3	Conj: hour 9	Anlagen, OM	Anlagen periphery; co‐localizes with Pdd1	Y	Nikiforov et al. (2000)
Cdl1	Conj: hour 9	Anlagen, OM, mac	Anlagen foci; weak mac	X	S. Horrell and D. Chalker, unpublished data
Cdl2	Conj: hour 6	NA^c^	NA	S	
Cdl3	Gr; conj: hour 15	NA^c^	NA	Z, S	

a Phase of lifecycle showing expression (mitotically growing = “Gr”; conjugation = “Conj”) and peak of expression in conjugation = “hour x”.

b mac = Macronucleus; mic = micronucleus; OM = old macronucleus.

c NA = not available (analysis was not done).

**Figure 3:**
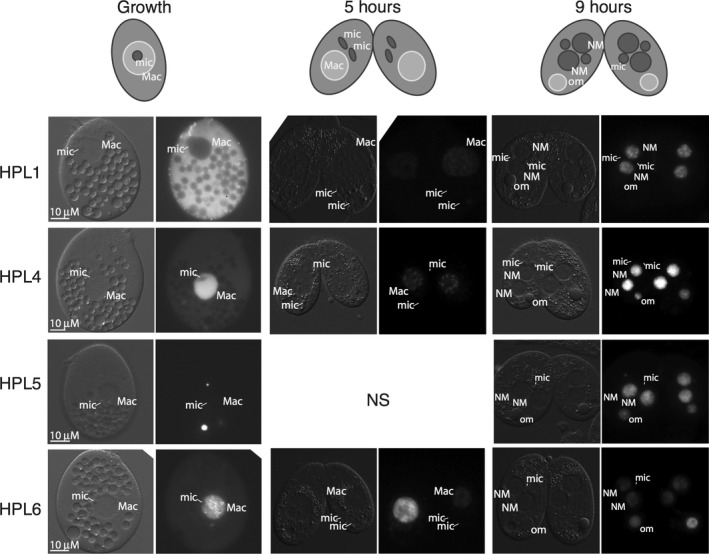
Hpl proteins localize to macronuclear chromatin, new developing macronuclei, and pycnotic nuclei. YFP fusion proteins were expressed by induction with CdCl_2_ and visualized by epifluorescence microscopy during regular growth, 5 h into conjugation, and 9 h into conjugation. Schematic at the top shows arrangements of various nuclei. DIC images on the left of each image pair show positions of nuclei. NS = not shown; NM = new macronucleus; mic = micronucleus; Mac = macronucleus; om = old macronucleus. Scale bars = 10 μm apply to all panels in the set.

Examination of YFP signal for each tagged protein revealed distinctive localization patterns. Neither Hpl1‐YFP nor Hpl5‐YFP localized to nuclei in growing cells. Hpl1‐YFP accumulated in the cytoplasm and exhibited an organized cortical pattern consistent with localization near basal bodies. These patterns were markedly different than that of Hpl5‐YFP, which was concentrated in a small number of cytoplasmic foci (Fig. 3). Neither *HPL1* nor *HPL5* is expressed at detectable levels during growth (Xiong et al. 2013), thus this lack of nuclear localization may indicate a requirement for a specialized nuclear import pathway for these proteins not present in growing cells. Though *HPL4* is also at undetectable levels in growing cells, Hpl4‐YFP exhibited diffuse accumulation in macronuclei, which is distinctly different from its closest homolog, Hpl5. *HPL6* is the only one of these first four genes whose expression is detected in growing cells (Xiong et al. 2013), and Hpl6‐YFP was the only one that showed macronuclear localization in subnuclear foci. These foci are qualitatively similar to foci formed by GFP‐Hhp1 and suggest chromatin association (Yale et al. 2016). These distinctive localization patterns observed are consistent with the hypothesis that each of these proteins have specialized functions.

To further probe the possible differentiation of these Hpl proteins, we expressed each YFP fusion in mating cells. During conjugation, parental macronuclei remain transcriptionally active until postzygotic development (starting ≥ 7 h post mixing) and new macronuclei differentiate from zygotic nuclei formed by the fusion of micronucleus‐derived gametic nuclei of each mating partner. In prezygotic (~5 h post mixing), Hpl5‐YFP remained absent from nuclei (not shown); in contrast, Hpl1‐YFP and Hpl4‐YFP now exhibited accumulation in small foci in parental macronuclei indicative of chromatin association. Hpl6‐YFP remained localized in parental macronuclei, but enriched in less defined subnuclear foci (Fig. 3). In postzygotic development (9 h postmixing), all four proteins, including Hpl5, accumulated within newly differentiating macronuclei, either in small foci (Hpl1) or enriched in specific subnuclear regions (Hpl4, 5, and 6) (Fig. 3). In addition, Hpl4, 5, and 6 remained localized in parental macronuclei, which are pycnotic at this stage. Although localization data is insufficient to define specific roles for these proteins, the distinct behavior of these homologous proteins is consistent with diversification of function and reveals differential means of protein accumulation and/or nuclear import.

**Figure 4:**
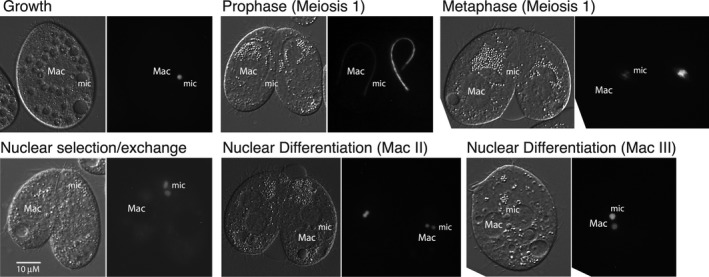
Hpl7 specifically targets chromatin in the germ line micronucleus. Hpl7‐YFP was expressed by induction with CdCl_2_ and visualized by epifluorescence microscopy during regular growth, and stages of meiosis and nuclear differentiation. Representative images are shown. The DIC images to the left in each pair show positions of nuclei. Mac = macronucleus; mic = micronucleus. Scale bar = 10 μm applies to all panels in the set.

Examination of Hpl7‐YFP underscores the functional differentiation of *Tetrahymena* Hp1‐like proteins. In contrast with the four HP1 homologs examined above that localized within macronuclei, Hpl7 localized specifically to micronuclei during both growth and development (Fig. 4). During meiosis, when chromosomes are extended (during prophase) or condensed (metaphase), it was apparent that Hpl7‐YFP is associated with chromatin. At the period of nuclear exchange, Hpl7 remained in gametic nuclei, but appeared to be rapidly lost from the meiotic products that are targeted for degradation. Hpl7 also specifically accumulated in new micronuclei during post‐zygotic development. Although Pdd1 localized to micronuclei during meiosis (Coyne et al. 1999), Hpl7 is unique among the HP1 homologs in its exclusive localization to micronuclei throughout the life cycle.

### Some chromoshadow domains influence protein localization

**Figure 5:**
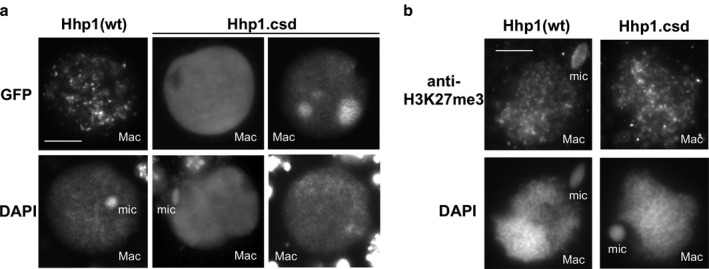
Loss of the CSD reduces chromatin body targeting of Hhp1 in nuclei from growing cells. (**a**) Cells were induced to express GFP‐Hhp1Δcsd (“Hhp1.csd”), then stained with DAPI and visualized by epifluorescence microscopy. Two sets of nuclear images representing two observed localization patterns of GFP‐Hhp1Δcsd are shown. Scale bar = 5 μm shown in first panel applies to all images. (**b**) Cells expressing GFP‐Hhp1Δcsd were subjected to immunofluorescence with anti‐H3K27me3, counterstained with DAPI, and visualized by epifluorescence microscopy. Mac = macronucleus; mic = micronucleus. Scale bar = 5 μm shown in first panel applies to all images.

**Figure 6:**
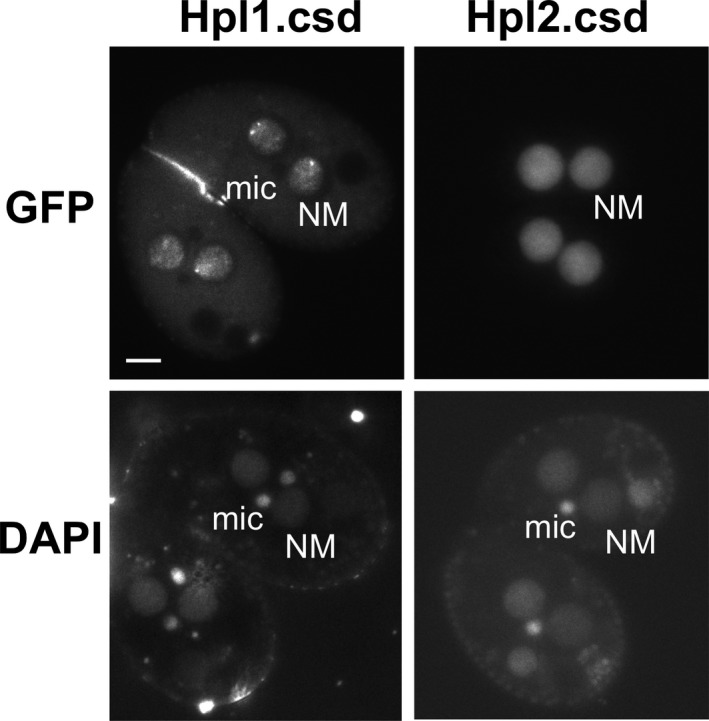
Loss of the CSD affects localization of Hpl2, but not Hpl1. Cells were induced to express GFP‐Hpl1Δcsd (“Hpl1.csd”) or GFP‐Hpl2Δcsd, then stained with DAPI and visualized by epifluorescence microscopy. NM = new macronucleus; mic = micronucleus. Scale bar = 10 μm shown in first panel applies to all.

A defining feature of a full HP1 protein is the presence of a CSD, which influences chromatin localization through homo‐dimerization and interaction with other proteins (reviewed in Eissenberg and Elgin Sarah 2014). Mutation of the CSD of Pdd1 prevented formation of Pdd1 foci in developing anlagen, suggesting that this domain is necessary for proper assembly of H3K9/27me‐marked heterochromatin into DNA elimination structures during nuclear development (Schwope and Chalker 2014). Of the three other proteins with CSDs, Hhp1 has the strongest CSD sequence similarity, yet it is classified as having a CD more similar to those on Pc proteins in other organisms, which typically do not contain CSDs. We thus tested whether the Hhp1 CSD influences chromatin targeting. N‐terminal fused GFP‐Hhp1 lacking a CSD (Hhp1.csd) appeared to be more uniformly distributed throughout the macronucleus instead of in small distinct foci—the expected pattern indicating normal heterochromatin body localization (Yale et al. 2016). In more than 50% of these cells, a few very large foci were evident instead (Fig. 5a). More uniform Hhp1 distribution might be caused by delocalization of H3K27me3 from chromatin foci in the cells expressing Hhp1.csd, but immunofluorescence with anti‐H3K27me3 antiserum showed that the H3K27me3 foci were still present in these cells (Fig. 5b). Thus, failure of GFP‐Hhp1.csd to form foci may have been caused by inability to stably target H3K27me3 chromatin foci. Together, these results suggest that the CSD normally participates in Hhp1 association with H3K27me3‐marked chromatin. To test whether the more divergent CSDs on two other proteins (Hpl1 and Hpl2) influence their localization, the CSDs were deleted by making C‐terminal truncations (refer to Fig. 1a for position of CSDs). N‐terminal GFP fusions of the truncated genes were expressed in wild type *Tetrahymena*. Similar to wild type Hpl1 (Fig. 3), GFP‐Hpl1.csd localized to chromatin foci in developing new macronuclei (Fig. 6) during the time period of its normal peak expression—hour 8–9 in conjugation (Xiong et al. 2013). However, the Hpl2 CSD truncation (GFP‐Hpl2.csd) was uniformly distributed across chromatin in developing new macronuclei, instead of in distinct foci (Xu et al. 2015). Deletion of the CSD from each of these CD proteins has distinctive effects on the proteins localization and is different than what was observed with truncation of Pdd1, which resulted in a defect in nuclear import (Schwope and Chalker 2014). Together our findings support extensive functional divergence among these chromatin regulatory proteins.

## Discussion

We describe an expanded and diversified family of thirteen HP1‐like proteins in *T. thermophila* that contain six of the 26 CD subtypes all grouping within an early‐diverging clade. The most N‐terminal CDs on all thirteen proteins contain the highly conserved caging amino acids required for methyl‐lysine binding, so are assumed to have chromatin binding activity. Four “full length” HP1 proteins contained both a CD and CSD, a number comparable to that in other eukaryotes with the greatest number reported as five in some *Drosophila* species (Levine et al. 2012). Including nine other *T. thermophila* “partial” HP1 proteins that possess the canonical HP1 CD motif but lack a CSD, the total of thirteen is large in comparison to other species analyzed accordingly (Levine et al. 2012).

### Specialization of *Tetrahymena* HP1‐like proteins may include differential histone binding

In *Drosophila,* some HP1 proteins such as HP1c associate with euchromatin, but it is only those with roles in heterochromatin function that have undergone pronounced expansion and diversification across species (Levine et al. 2012). *Tetrahymena* heterochromatin takes several forms each with specialized roles; notably the micronuclear genome is packaged in condensed chromatin throughout most of the cell cycle, DNA destined for elimination from developing macronuclei is found in heterochromatin enriched in H3K9me2/3, and a significant portion of the DNA within the transcriptionally active macronucleus resides in heterochromatin bodies. Our data showing diversification of HP1‐like proteins in this model system is consistent with the idea that evolution of functionally distinct HP1‐like proteins encoded in the genome may have been driven by the need to differentially regulate distinct heterochromatin states (Levine et al. 2012). For example, we noted CD amino acid sequence differences that, together with localization data, indicate CD protein specialization for differentially methylated heterochromatin. In *Tetrahymena*, heterochromatin in mature nuclei lacks methylated H3K9 and instead is marked with H3K27me3. Proteins from *Drosophila* and humans that bind H3K27me3 tend to be more basic than H3K9me2/3 binders (Kaustov et al. 2011). Consistent with this, the strongest CDs of four out of five *Tetrahymena* proteins that localize to mature macronuclei marked with methylated H3K27 were among the most basic in character (Hhp1, Hpl6, Hpl4, Cdl1), while those expressed only in conjugation and that make foci in developing nuclei containing methylated H3K9 were notably more acidic (Table 3, Fig. 3). In addition to a basic CD, two of the macronuclear‐localizing proteins are normally expressed only during vegetative cell growth (Hhp1 and Hpl6) and these both possessed nonpolar “clamp” residues that typify H3K27me3 binding domains. In contrast, the two amino acids that form a polar “clamp” typical of H3K9me2/3 binders were detected on the majority of proteins expressed exclusively during conjugation, and those tested localize to anlagen at a time when H3K9 methylation occurs (Fig. 2, 3 and Table 4). Two of these, Pdd1 and Pdd3, which contain both polar clamps and acidic CDs are known to bind H3K9me2 (Taverna et al. 2002). In most cases the HP1‐like proteins examined had CD amino acid sequence acidity/basicity and polar clamp features that reflected their expected histone binding specificity based on the available histone modifications on chromatin where they localize. Exceptions, such as Hpl7, were more neutral and/or lacked a pair of polar or nonpolar clamp residues (Table 3 and Fig. 2). In all, these results suggest that the same amino acid sequence features that correlate with histone binding specificities of *Drosophila* and human CD proteins may also be suggestive of histone binding activity of *Tetrahymena* CD proteins.

In another example of specialization, at least four of the five proteins that contained a second less conserved CD (subtype S) associated with chromatin foci in developing macronuclei—presumed DNA elimination foci (Fig. 1a, 3 and Table 4). Two CDs is a feature of the CHD family of proteins where both domains cooperate to bind methylated H3K4 (Flanagan et al. 2005). However, all second CDs in the *Tetrahymena* proteins lacked a full complement of caging amino acids suggesting that they were subjected to more rapid mutation than the primary CDs and have lost methyl‐binding activity (Fig. 2). It is possible that second CDs in putative DNA elimination proteins have evolved new specialized functions, or simply that these proteins do not require the chromatin binding activity of two CDs.

### Germ line specialization

Out of ten *Tetrahymena* proteins analyzed for cellular distribution, only one (Hpl7) localized to the highly condensed, transcriptionally inert chromatin in the germ line micronucleus. This CD‐only protein resided exclusively with micronuclear chromatin throughout all stages of the life cycle (Fig. 4). *HPL7* is expressed at relatively low levels during vegetative cell growth but exhibits dynamic expression changes coincident with a brief period of micronuclear transcription and meiosis (Xiong et al. 2013) (Fig. 4). Similar CD‐only HP1‐like proteins are known in *Drosophila*. Derived from HP1D/Rhino, which promotes piRNA production for transposon‐silencing genome defense only in female germ line cells (Klattenhoff et al. 2009; Vermaak et al. 2005), these CD‐only genes were also expressed predominantly in germ line cells (Levine et al. 2012). Whereas *Drosophila* species express two or more germ‐line CD proteins, *Tetrahymena* appears to express only one. *HPL7* presents another example of *Tetrahymena* CD protein specialization for specific chromatin functions, in this case a germ line specific function that may involve global chromosome compaction. Our sequence analysis of the chromo domain suggests that Hpl7 might preferentially bind H3K9me2/3, but this modification is lacking from germ line micronuclei in which it resides. Instead, these nuclei contain H3K27me3 and for a 4‐h period covering meiosis, are additionally marked with H3K27me3, which is necessary for protecting heterochromatin from double‐strand breaks (Papazyan et al. 2014). We speculate that Hpl7 has evolved to interact with H3K27me3 throughout the life cycle, and we raise the possibility that these interactions may be influenced by the presence of H3K23me3 on the same nucleosomes.

### Specialization for genome rearrangement and DNA elimination

More than half (eight) of the CD proteins studied are expressed exclusively during conjugation. These eight have amino acid sequences that are more typical of H3K9Me binders, and they localize to developing macronuclei after H3K9me2 is established, and before this modification is removed from the genome along with the IESs. Four of these are required for or otherwise linked to DNA elimination (Coyne et al. 1999; Nikiforov et al. 2000; Taverna et al. 2002; Woehrer et al. 2015; Xu et al. 2015). It is thus tempting to speculate that these proteins evolved to ensure the accuracy and efficiency of heterochromatin elimination in this ciliate. Within this group of proteins, our localization data suggests that these CD proteins have taken on specialized roles during genome reorganization. A good example of such diversification can be observed in the behavior of Hpl4 and Hpl5. Their CDs (both type L) have a strong phylogenetic relationship (Fig. 1c), and the genes encoding these two closely related CD proteins are arranged in tandem in the genome; a configuration that presumably arose by gene duplication event. Hpl4/5 proteins are nearly identical in size (Fig. 1a), and are expressed at the same life cycle stages. Despite these similarities, they have sufficient differences that appear to indicate differential functions. Hpl4 has a second CD, whereas Hpl5 does not, indicating that after gene duplication the second CD on Hpl5 was subjected to more rapid mutation. Differential protein properties are evident from localization data: when expressed from the same vector, only Hpl4 accumulated in macronuclei of cells that were growing or in pre‐zygotic development (Fig. 3, “Growth” and “5 h”) while Hpl5 failed to accumulate in nuclei prior to development of the anlagen nuclei (Fig. 3, “9 h”). Although both proteins localized to anlagen nuclei coincident with their peak expression at 8 h in conjugation, Hpl4 accumulated in foci whereas Hpl5 showed more uniform distribution in these nuclei.

We expressed the HPL‐fluorescent protein fusions by ectopic expression from a replicating vector. Even so, we observed distinctive localization patterns, suggesting that some functional diversification is regulated through the import of these proteins into the correct nuclei at the proper development stage. This point can be readily observed comparing the accumulation of the closely related Hpl4 and 5 expressed in growing cells (Fig. 3). Hpl4 accumulated in the nucleus whereas Hpl5 was observed in a few aggregates in the cytoplasm. These differences might be explained by nuclear import mechanisms as *Tetrahymena* encodes large families of nuclear localization receptors (importin alpha and beta) that have different expression and localization patterns (Malone et al. 2008). Once within the proper nucleus, the sub‐genome organization appears to be distinct for each protein, further supporting functional differences between these related proteins.

### Degradation of chromo shadow domain function in *Tetrahymena*

Differences in the CD proteins studied extended beyond their CD sequence/putative binding affinities. Chromo shadow domains, which exhibit extensive diversity, were recognizable on only four of the thirteen proteins analyzed. CSDs are known to participate in localizing HP1 proteins to H3K9me2/3, something demonstrated by the CSD on *Tetrahymena* Pdd1, which is required for its localization to nuclear foci (Schwope and Chalker 2014). We were intrigued by Hhp1: it possesses a CD with characteristics more similar to the Pc than HP1 family but also contains the CSD of HP1 proteins, which is absent from Pc homologs (Yale et al. 2016). This may suggest that the differentiation of HP1 and Pc proteins occurred after the divergence of ciliates and organisms that have Pc. Our data show that the CSD is necessary for normal Hhp1 localization to chromatin bodies in the macronucleus typically marked with methylated H3K27 (Fig. 5), similar to its function localizing HP1 homologs to methylated H3K9. This is an example of a protein that has evolved hybrid characteristics of two CD protein families. Among proteins that have methylated H3K9 binding characteristics, the CSD on Hpl2 appeared necessary for the normal punctate sub‐localization in developing anlagen nuclei (Fig. 6); (Xu et al. 2015), but this same domain did not appear necessary for similar sub‐localization of Hpl1 in the same set of nuclei. This could indicate degradation of the CSD function, which appeared to typify the *Tetrahymena* HP1‐like proteins as the majority lacked a recognizable CSD.

Examples of expansion and diversification of the HP1 family have been found in other species—the fifteen CD‐containing homologs in *Drosophila* and at least three in humans and mouse (Hp1α/β/γ), all of which show specialization through differential localization to functionally distinct chromatin domains. *Tetrahymena*, with heterochromatin specialized for DNA elimination, whole genome condensation, and gene silencing, expands the palette of examples of HP1 family expansion and diversification. Further study to discover the individual roles each of these related proteins play promises to provide more nuanced insights into the diversity of structures and related functions of what is broadly defined as heterochromatin.
